# A Model for Infrastructure Detection along Highways Based on Remote Sensing Images from UAVs

**DOI:** 10.3390/s23083847

**Published:** 2023-04-10

**Authors:** Xian Jiang, Qing Cui, Chongguo Wang, Fan Wang, Yingxiang Zhao, Yongjie Hou, Rujun Zhuang, Yunfei Mei, Gang Shi

**Affiliations:** School of Information Science and Engineering, Xinjiang University, Urumqi 830017, China

**Keywords:** remote sensing, highway infrastructure, intelligent roads, UAVs, object detection

## Abstract

Infrastructure along the highway refers to various facilities and equipment: bridges, culverts, traffic signs, guardrails, etc. New technologies such as artificial intelligence, big data, and the Internet of Things are driving the digital transformation of highway infrastructure towards the future goal of intelligent roads. Drones have emerged as a promising application area of intelligent technology in this field. They can help achieve fast and precise detection, classification, and localization of infrastructure along highways, which can significantly enhance efficiency and ease the burden on road management staff. As the infrastructure along the road is exposed to the outdoors for a long time, it is easily damaged and obscured by objects such as sand and rocks; on the other hand, based on the high resolution of the images taken by Unmanned Aerial Vehicles (UAVs), the variable shooting angles, complex backgrounds, and high percentage of small targets mean the direct use of existing target detection models cannot meet the requirements of practical applications in industry. In addition, there is a lack of large and comprehensive image datasets of infrastructure along highways from UAVs. Based on this, a multi-classification infrastructure detection model combining multi-scale feature fusion and an attention mechanism is proposed. In this paper, the backbone network of the CenterNet model is replaced with ResNet50, and the improved feature fusion part enables the model to generate fine-grained features to improve the detection of small targets; furthermore, the attention mechanism is added to make the network focus more on valuable regions with higher attention weights. As there is no publicly available dataset of infrastructure along highways captured by UAVs, we filter and manually annotate the laboratory-captured highway dataset to generate a highway infrastructure dataset. The experimental results show that the model has a mean Average Precision (mAP) of 86.7%, an improvement of 3.1 percentage points over the baseline model, and the new model performs significantly better than other detection models overall.

## 1. Introduction

In recent years, intelligent roads have become an industry research hotspot, and the construction of intelligent roads can effectively improve the capacity of existing road networks. As one of the important assets, exploring the dynamic management of road infrastructure through 3D real-world and other technologies is particularly important for the construction of intelligent roads. In China, the construction of road infrastructure is moving towards digitalization as the total mileage of roads continues to increase and the demand for management and maintenance grows [1]. Various factors need to be taken into account in the construction of digital infrastructure, including traffic flow [2], road conditions, types of vehicles, extra-large cargo movements [3], and so on. All these factors affect the construction and maintenance of the infrastructure.

In the early days, the statistical work of infrastructure along the highway was performed using a manual survey, that is, the highway staff carried out an on-site survey to count the types and locations of infrastructure to provide data support for the subsequent management and maintenance work. However, the manual data collection method was inefficient, required a lot of human and material resources, was prone to omissions and statistical errors, and brought about problems, such as the compromised personal safety of staff. With the advantages of high efficiency, low cost, a wide field of view, flexibility, and safety, UAV imagery is widely used in military, transportation, agriculture, search and rescue, and other fields [4]. There is therefore an urgent need to explore efficient and non-destructive techniques for the task of data collection on infrastructure along highways. We have performed research related to the infrastructure of target detection algorithms in UAV imagery for automatic detection and classification tasks.

Target detection is now well established in natural scenes, such as face recognition, vehicle detection and traffic sign recognition, but the data used for this type of task is captured horizontally by collectors, such as vehicle-mounted cameras. On the other hand, the shape, size, color, and general appearance of the infrastructure in the images taken by UAVs may change significantly, for example, the sizes of target objects vary greatly, and culverts and barriers take up many more pixels than objects such as warning stakes and milestones, resulting in unsatisfactory detection results when existing target detection algorithms are directly applied to high-definition UAV aerial images, and there are also more missed and false detections of small objects. It is of great significance to study the detection and classification of infrastructure along highways based on UAV images; however, it is difficult in this research to enable the detection model to effectively use the feature information of small targets to improve the detection capability of small targets.

The main contributions of this study are as follows:

- An improved anchor-free multi-scale target detection method, MSA-CenterNet based on CenterNet [5], is proposed;
- To better detect various types of infrastructures of different sizes in UAV images, we introduce a multi-scale feature fusion module to enhance the feature extraction capability of the network model and obtain more contextual semantic information;
- Due to the high proportion of small targets, such as warning stakes, milestones, and traffic signs in the dataset, an attention mechanism is used to enrich the semantic information of the feature map while enhancing the feature representation capability;
- A high-resolution infrastructure dataset along the highway is obtained through processing such as UAV photography and filtering and labeling to provide basic training data.

The paper is organized as follows: Section 2 presents the progress of research on target detection and UAV-image-based target detection algorithms for agricultural, industrial and rescue applications; Section 3 details the proposed improved method based on the CenterNet model; Section 4 presents the experimental results of the comparison and ablation experiments; and Section 5 concludes the work of this paper.

## 2. Related Works

A major feature of traditional target detection algorithms is the use of human-designed features, such as scale-invariant feature transform (SIFT) [6] and histogram of orientation gradients (HOG) [7], Hinton’s team proposed AlexNet [8] using convolutional neural networks (CNN) to extract the deeper features of images for target detection and classification. There are currently two main types of deep-learning-based target detectors: (1) single-stage target detectors, which directly regress the class probability and location of objects through the backbone network without generating a region of interest (ROI), such as the YOLO series [9,10,11,12] and SSD and Retina-Net [13,14], which have been widely used in the direction of target detection for UAV images as the detection accuracy and speed of such detectors continue to break through. (2) The other is a two-stage target detector: the first step has to be first into a region of interest proposal, then the second step is for each region proposal and bounding box regression for object classification. These include common detection models such as Faster R-CNN [15], which improves detection ability by generating regions of interest (ROI) with a region proposal network (RPN); however, the Faster R-CNN uses only the last layer of features in the prediction stage and ignores the features of other layers, resulting in the poor detection of small targets. Cascade RCNN [16] used the second stage of Faster R-CNN in a cascade manner and set different thresholds for positive and negative samples to improve the detection accuracy of small targets. The current direction in which target detection is more widely used in traffic is the detection and recognition of traffic signs: Jeziorek et al. [17] proposed a method for traffic sign detection and recognition based on event camera information, which to some extent alleviates the problems of low dynamic range and difficulty in working properly under illumination conditions that exist with standard frame cameras; Manzari et al. [18] proposed a new pyramidal Transformer with a local mechanism to achieve the detection of traffic signs, overcoming the problems of small dataset size and uneven category distribution.

Drones are increasingly being used in both civilian and military applications. The potential for mission failure is high in both civilian and military applications due to the visual degradation of aerial imagery captured by UAVs [19]. Velusamy et al. [20] point out the challenges of drones in crop and pest management in agriculture; Lygouras et al. [21] proposed a method for conducting search and rescue missions using real-time human detection by UAVs; Almagbile [22] proposed an accelerated segmentation test (FAST)-based algorithm to detect crowd features in UAV images taken with different camera orientations and positions. Compared to other target detection areas, UAV imagery for infrastructure along highways is scarce in the literature, with the HighD [23] dataset for vehicle detection being the main one currently publicly available; Regarding VisDrone [24], a dataset containing targets such as pedestrians, cars, and bicycles, which was collected by AISKYEYE, a machine learning and data mining laboratory at Tianjin University, Vaddi et al. [25] proposed an end-to-end target detection model on this dataset using ResNet and MobileNet as convolution for real-time applications on a UAV platform. The research on the target detection of facilities by UAV images in traffic is still at an early stage, and Tang et al. [26] proposed an improved detection method based on Faster R-CNN using a super region proposal network (HRCNN) to verify the candidate regions and improve the vehicle detection accuracy. Moreover, Duan et al. [27] explored a method of quickly acquiring the target images of the central green belt of a highway using a quadrotor UAV and digital image processing technology, and Li et al. [28] presented a study on the application of UAV and IoT technology in the intelligent inspection of bridge engineering and the appearance inspections of bridge engineering by UAV.

With the continuous development of deep learning techniques, there have been breakthroughs in the precision and speed of target detection algorithms. The popularity of UAVs has opened up a new research area for target detection, attracting the attention of many scholars. However, due to the wide perspective, complex backgrounds, and high proportion of small targets in UAV aerial images, the general target detection algorithm based on deep learning cannot achieve good results when applied directly to UAV aerial images. Therefore, there is a need to conduct targeted algorithm research on the detection of infrastructure along roads from UAV aerial photography.

## 3. Proposed Method

**Figure 1:**
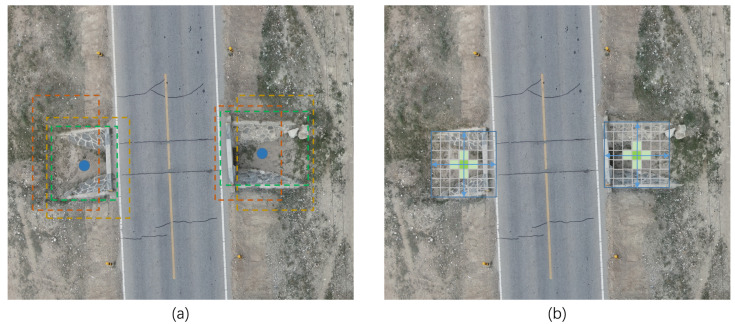
(**a**) The anchor-based algorithm uses anchors to extract candidate target frames, and the quality of the anchor affects the detection of the model and introduces additional non-maximal suppression (NMS) calculations. The blue dot in (**a**) indicates the target center point, and the rectangular boxes of different colors indicate anchor boxes of different qualities, with the better the fit with the target, the better the quality of the anchor box, among which the green box has the best effect. (**b**) The anchor-free algorithm generates the center points of the targets directly.

Our proposed algorithm uses CenterNet as the base model to locate the center point of the target to be detected by key point estimation, represent that target by its center point, and regress other attributes of the target (multiple attributes corresponding to multiple channels), such as size, 3D positions, orientation, and even pose, directly from the image features. Compared to detection network models such as the YOLO series, SSD, Faster R-CNN and RetinaNet which rely on many predefined anchor boxes, our improved method is still an anchor-free target detection model, which does not require a fixed set of anchor boxes to be preset in the prediction stage but predicts the centroid position and class of each target directly from the feature map. Compared with the anchor-based approach, the anchor-free approach does not rely on a priori knowledge and does not require the classification and regression of candidate boxes. This can simplify the detection process and improve the efficiency and accuracy of detection. The difference between the anchor-based and anchor-free detection models is illustrated in Figure 1.

### 3.1. Backbone Network Overview

MSA-CenterNet uses an anchor-free-based approach to locate the center point of a target directly, dividing the task of target detection for infrastructure along a road into three main steps: Firstly, the image is pre-processed by scaling; secondly, feature extraction is performed by the backbone network to obtain a preliminary feature map; thirdly, the feature map is upsampled by deconvolution and passed to the three branching convolutional networks for prediction. A heatmap is generated for each target. The peaks in this heatmap correspond to the center points of the target objects to be detected, and the image features predict the width and height properties of the object bounding box at each peak.

**Figure 2:**
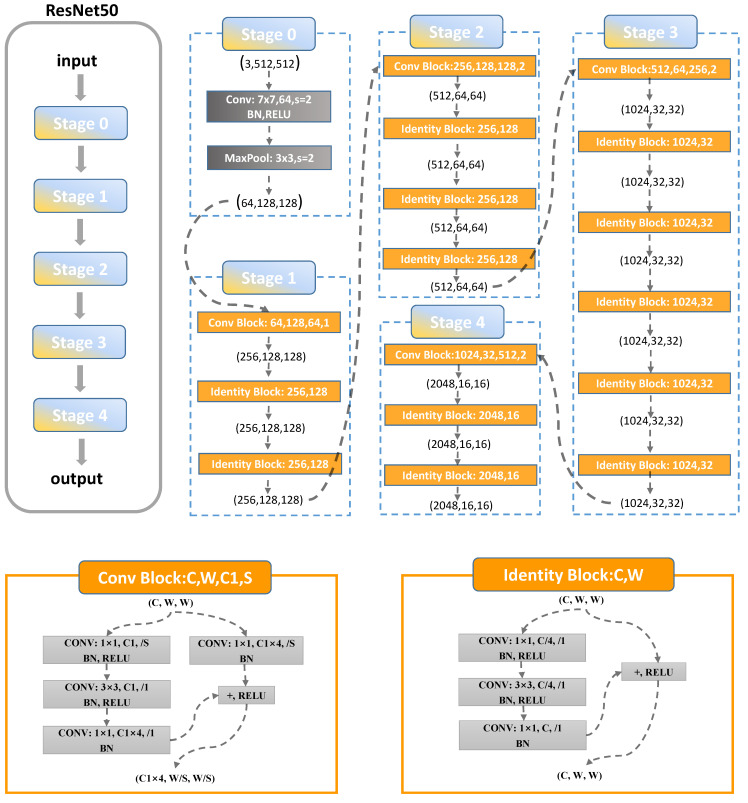
The ResNet50 network structure.

To achieve both accuracy and speed, we adopt ResNet50 as the backbone network for extracting features from images, and a deep network for obtaining deep semantic features in UAV remote sensing images, as shown in Figure 2. In ResNet residual networks, instead of fitting the original mapping directly, a residual mapping is used to fit the residual mapping. This way, the network can learn more effectively. A residual network can be understood as adding shortcut connections to the forward network, which skip some layers of data output and pass the original data directly to the input part of the subsequent data layers. A 1×1 convolution is used to compress and expand the feature map channels, replacing the original two 3×3 convolutional residual blocks. This ensures that the added shortcut connections do not increase the parametric count and complexity of the model but rather speed up the forward inference of the network. This allows the content of the later feature layer to be partially contributed by one of its preceding layers linearly.

ResNet50 uses the bottleneck module with 1×1, 3×3, and 1×1 convolution. The 1 × 1 convolution kernel first reduces the number of channels in the image from 256 to 64, then does a 3×3 convolution on the 64-channel-count feature map, and finally scales the number of channels by 4x with 1×1 convolution. The use of the bottleneck structure can effectively reduce the parameters of the model. Overall, the number of channels becomes twice as large after this module.

### 3.2. Multi-Scale Feature Fusion Module

However, the ResNet50 module alone does not solve our problem well, as the most challenging problem in target detection is the problem of the scale variation of the target. In the dataset we have constructed, there are large differences in the shape and size of the objects, such as the relatively large size of the culvert category in the target to be detected, while the alarm posts take up a small number of pixels and there are cases where the camera angle is extreme (vertical shots), with even objects of extremely small or extreme shapes (e.g., elongated roadside barriers) presenting a significant challenge in terms of accurate target detection and precise localization.

Among the existing algorithms proposed for the problem of large variations in target size, the more effective ones are feature pyramids [29] and image pyramids, both of which use multi-scale feature extraction and fusion to detect objects of different sizes. The feature pyramid network takes advantage of the fact that targets of different sizes have different features and uses shallow and deep features to distinguish between targets, solving the problem of small-target information loss during down sampling; however, the feature pyramid network generates more extra convolutional computations and parallel branches, resulting in slower inference. Image pyramids, on the other hand, are collections of images consisting of multiple sub-images of an original image at different resolutions, obtained by stepped-down sampling at different scales; however, this method severely increases the computational effort of the model.

Target detection networks use convolutional neural networks to extract features of the target to be detected by means of layer-by-layer abstraction, where an important concept is the perceptual field. We can learn that the high-level network has a relatively large perceptual field and strong representation of semantic information; however, its problem is the low resolution of the feature map, weak representation of geometric information, and a lack of spatial geometric feature details. The low-level network, in contrast, has a relatively small perceptual field, strong representation of geometric detail information, and a weak representation of semantic information despite high resolution.

**Figure 3:**
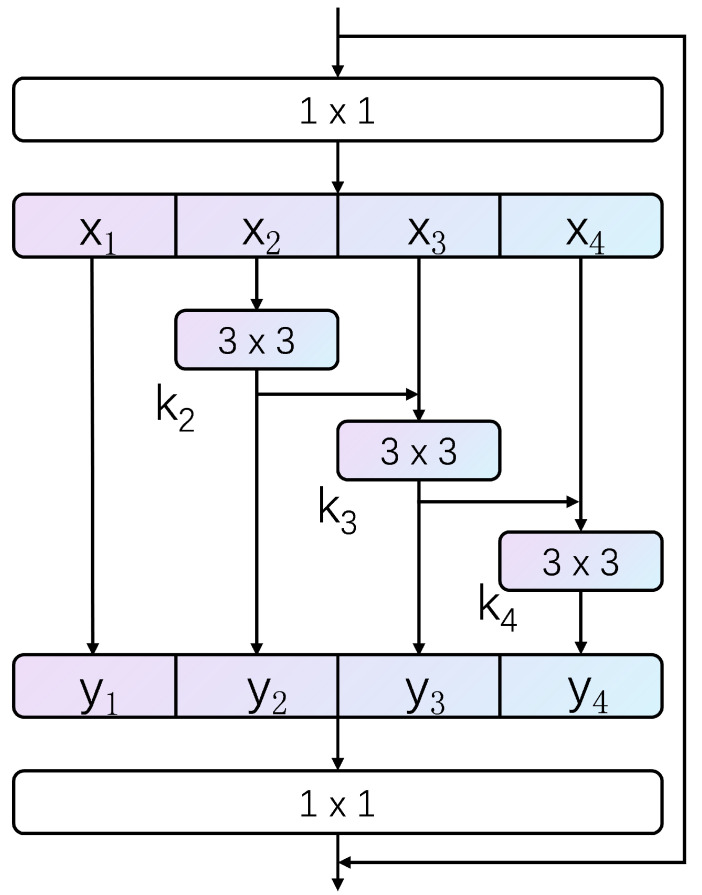
The structure of the feature fusion module.

In this paper, the Res2Net [30] building block is used to replace the bottleneck module in ResNet50, and the Res2Net feature fusion layer is shown in Figure 3. Improving the detection capability of the model by increasing the perceptual field within a block rather than capturing images at different scales layer by layer at a finer granularity means that a similar residual connection with a hierarchy is constructed within the original single residual structure, and this residual feature fusion layer is used to expand the size of the perceptual field and produce a fine-grained feature map without destroying the coarse-grained features.

First, we adjust the input channels to 512 by a 1×1 convolutional layer without considering the backbone network. We then divide the feature maps of the N channels (here N = 512) equally into s groups, with each subset of the feature maps denoted by $x_{i}$, where i∈{1,2,3,…,s}, and based on the experience of previous studies, s is taken to be the classical value of 4, which makes the channels divisible and enables the appropriate size to be obtained. Each feature map subset $x_{i}$ has the same spatial size, and the number of channels per channel is equal to N/s. Each channel, except $x_{1}$, is convolved through a 3×3 convolution layer, represented by $K_{i}\left(\cdot \right)$, and the output of the convolution is represented by $y_{i}$; then, the output of $K_{i}$ is added to $K_{i+1}$ and fed into $K_{i+1}\left(\cdot \right)$. Each intermediate output $y_{i}$ is defined as follows:

(1) $$y_{i}=\left\{\begin{array}{cc} x_{i} & i=1;\\ K_{i}\left(x_{i}\right) & i=2;\\ K_{i}\left(x_{i}+y_{i-1}\right) & 2<i\leq s. \end{array}\right.$$

where $K_{i}$ denotes the convolution operation on inputs $x_{i}$ and $y_{i-1}$, where $y_{1}$ is identical to $x_{1}$, retaining the coarse-grained features of the 1×1 receptive field, $y_{2}$ obtains a 3×3 receptive field after a 3×3 convolution operation, $y_{3}$ obtains a 5×5 receptive field by performing a 3×3 convolution operation based on $y_{2}$, $y_{4}$ can further build on $y_{3}$ to obtain a larger 7×7 receptive field, so that outputs of different numbers and different sizes of receptive fields can be obtained, and finally the four outputs are stitched together and fused in the channel dimension, and the 1×1 convolution operation is then performed. This method of splitting first and then fusing allows for the effective acquisition of multi-scale features as well as better feature fusion.

### 3.3. Inverted Residual Attention Module

The attention mechanism is a data processing method for machine learning that is widely used in various types of machine learning tasks such as image processing and natural language processing. Research work on deep learning combined with attention mechanisms in computer vision has focused on the use of masks to form attention mechanisms that enable them to ignore irrelevant information and focus on key object feature information. The idea of the mask is to use another new layer of large weights, which are learned by training, to identify the key feature information in an image. This allows the deep neural network to learn which regions to pay attention to in each new image, thus forming attention.

**Figure 4:**
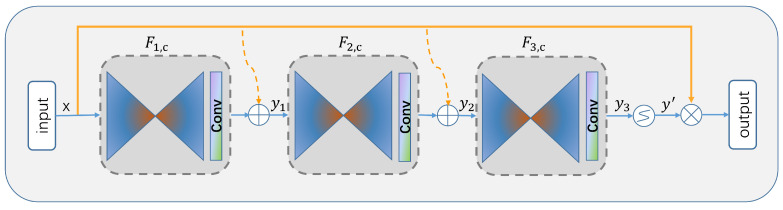
Inverted residual attention module structure.

In this paper, we further simplify and improve the residual attention network [31] by using a module that adds a mask mechanism to the soft attention based on the idea of a residual network [32]. The module needs to take not only the feature tensor of the current network layer after adding a mask to the information as input to the next layer but also the feature tensor before adding the mask as input to the next layer, which can effectively prevent the problem of not being able to stack the network layers very deep due to too little information after the mask, and provide richer features so that it can better focus on key features. The network structure is shown in Figure 4.

The inverted residual attention network consists of three attention modules, followed by a simple sigmoid-activation function to generate the attention weights, and finally a dot product operation with the input feature map to improve the local sensitivity of the feature map. By finding the corresponding attention weights for each feature element, both spatial-domain and channel-domain attention can be formed.These stacked residual modules are attention-aware, and the attention awareness of the different modules can change adaptively as the modules go deeper, so that different types of attention can be captured in different attention modules.

The output of each residual attention module is represented by $y_{i}$, expression (2), and i=1,2,…,N. In this paper, N is taken to be 3, indicating that three inverted residual attention modules are stacked.

(2) $$y_{i}=F_{i,c}\left(x\right)*\left(1+M_{i,c}\left(x\right)\right)$$

Specifically, in each attention module, starting from the input, the maximum pooling layer is first executed to expand the global information through a top–down architecture, and after obtaining low-resolution features, the output is passed to the next module after a full convolution operation to increase the perceptual field, while filtering out features with little role and redundant information, and retaining the role of key information. Where *x* is the input to the residual module, F(x) is a function of the three-layer convolution block, M(x) is the mask branch weight in the range [0,1], $y_{i}$ approximates the original feature F(x) as M(x) approaches 0, and $y_{i}$ is the temporary output feature after one residual unit. To allow for better preservation of the attention mapping, we only pass the last output state $y_{N}$ through a sigmoid-activation layer in the last part of the model, so that the attention weights fall in the range between 0 and 1. Because the attention mapping ranges from 0 to 1 element by element in this process, the introduction of multiple sigmoid-activation layers in the attention module would lead to an overall performance degradation. The attention weights are generated as follows:

(3) $$F\left(x_{i,c}\right)=\frac{1}{1+exp(-x_{i,c})},i=0,1,2,\ldots ,N.$$

We use Equation (3) to obtain mixed attention for each channel and spatial location using a sigmoid-activation layer, where better performance can be obtained by adaptively changing attention through features without any additional constraints. Where *i* covers all spatial locations on each channel and N denotes the feature map size, and c denotes the channel index, $x_{i,c}$ represents the attention weight generated by each channel at a specific spatial location.

### 3.4. Loss Function

The loss function of the MSA-CenterNet model consists of the KeyPoint loss $L_{k}$ for the heatmap, the target center point offset $L_{off}$, and the target size prediction loss $L_{size}$. For $L_{k}$, we use a modified pixel-level logistic regression focal loss, and $L_{size}$ and $L_{off}$ are trained using $L_{1}$ loss. The weights $\lambda_{size}$ are taken as 0.1, and $\lambda_{off}$ is taken as 1. The model loss function is calculated as in Equation (4).

(4) $$L_{det}=L_{k}+\lambda_{size}L_{size}+\lambda_{off}L_{off}$$

The target KeyPoint loss $L_{k}$ is calculated using a modified focal loss of pixel-level logistic regression as in (5), where α and β are hyperparameters, which are set to 2 and 4, respectively, in this experiment to equalize the hard and easy samples and the positive and negative samples.

(5) $$L_{k}=\frac{-1}{N}\sum_{xyc}\left\{\begin{array}{cc} {\left(1-{\hat{Y}}_{xyc}\right)}^{\alpha }\log({\hat{Y}}_{xyc}) & \mathrm{if}\;Y_{xyc}=1;\\ {\left(1-Y_{xyc}\right)}^{\beta }{\left({\hat{Y}}_{xyc}\right)}^{\alpha }\log\left(1-{\hat{Y}}_{xyc}\right) & otherwise. \end{array}\right.$$

N in Equation (5) indicates the number of key points in the image (i.e., the number of positive samples) and is used to normalize all positive focal loss to 1. The summation symbol subscript xyc indicates all coordinate points on all heatmaps (*c* indicates the number of target categories, and each category corresponds to a heatmap), and ${\hat{Y}}_{xyc}$ indicates the predicted value and $Y_{xyc}$ is the labeled true value.

The center point offset loss $L_{off}$ is calculated using $L_{1}$ loss, Equation (6), which is calculated for the offset value loss of positive samples only. As the image is down sampled, the key points of the ground truth are biased because the data are discreet, where ${\hat{O}}_{\tilde{p}}$ represents the predicted local offset value, *p* is the coordinate of the target centroid in the image, *R* is the scaling ratio, and $\tilde{p}$ is the approximate integer coordinate of the target centroid after scaling. The calculation is as follows:

(6) $$L_{off}=\frac{1}{N}\sum_{p}|{\hat{O}}_{\tilde{p}}-\left(\frac{p}{R}-\tilde{p}\right)|.$$

The object size prediction loss value $L_{size}$ is also calculated using $L_{1}$ loss for positive samples only. Let ($x_{1}^{\left(k\right)},y_{1}^{\left(k\right)},x_{2}^{\left(k\right)},y_{2}^{\left(k\right)}$) be the bounding box of object k with category $c_{k}$. Its center point is located at $p_{k}=\left(\frac{x_{1}^{\left(k\right)}+x_{2}^{\left(k\right)}}{2},\frac{y_{1}^{\left(k\right)}+y_{2}^{\left(k\right)}}{2}\right)$. ${\hat{S}}_{pk}$ is the prediction size and $s_{k}$ is the true size, and its size is $s_{k}=\left(x_{2}^{\left(k\right)}-x_{1}^{\left(k\right)},y_{2}^{\left(k\right)}-y_{1}^{\left(k\right)}\right)$. The calculation is as follows:

(7) $$L_{size}=\frac{1}{N}\sum_{k=1}^{N}|{\hat{S}}_{pk}-s_{k}|.$$

## 4. Experiments Section

In this paper, the proposed method is experimentally validated, and ablation experiments are conducted in order to demonstrate the improved multi-scale feature fusion module as well as the attention mechanism. In the comparative experimental part, the paper is conducted on home-made data with some of the mainstream algorithms, and the proposed method performs better in terms of detection precision and mean Average Precision (mAP), which can meet the requirements of industrial applications.

The training, testing and validation tasks were performed on an NVIDIA TITAN RTX graphics processing unit with 24 GB of video memory running the Ubuntu 18.04 operating system. For the experiments, using the ADAM optimizer, we set the batch size for model training to 32, with a default learning rate of 0.000125 and a weight decay of 0.0005, for 140 training periods.

### 4.1. Infrastructure Datasets along Highways

**Figure 5:**
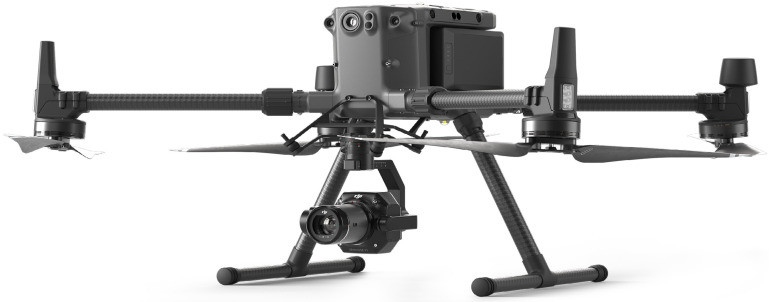
DJI ZenmuseP1 drone.

The dataset used for the experiments conducted in this paper was captured by our lab using a DJI ZenmuseP1 drone on a selected section of highway in Xinjiang, China, obtaining an image resolution of 8192×5460, and the spatial resolution of the drone images is 1 cm/pixel. The equipment used for aerial image capture is shown in Figure 5. We selected 1500 representative images containing infrastructure from the captured images for manual annotation, and divided them into a training set, a validation set, and a test set according to 8:1:1.

The open-source image annotation tool LabelImg [33] was used to annotate the infrastructure dataset along the highway, using rectangular boxes to mark a total of eight types of infrastructure such as culverts, traffic signs, warning stakes, barriers, and road curb signs in the images and convert them to coco dataset format. Table 1 describes the dataset in detail.

**Figure 6:**
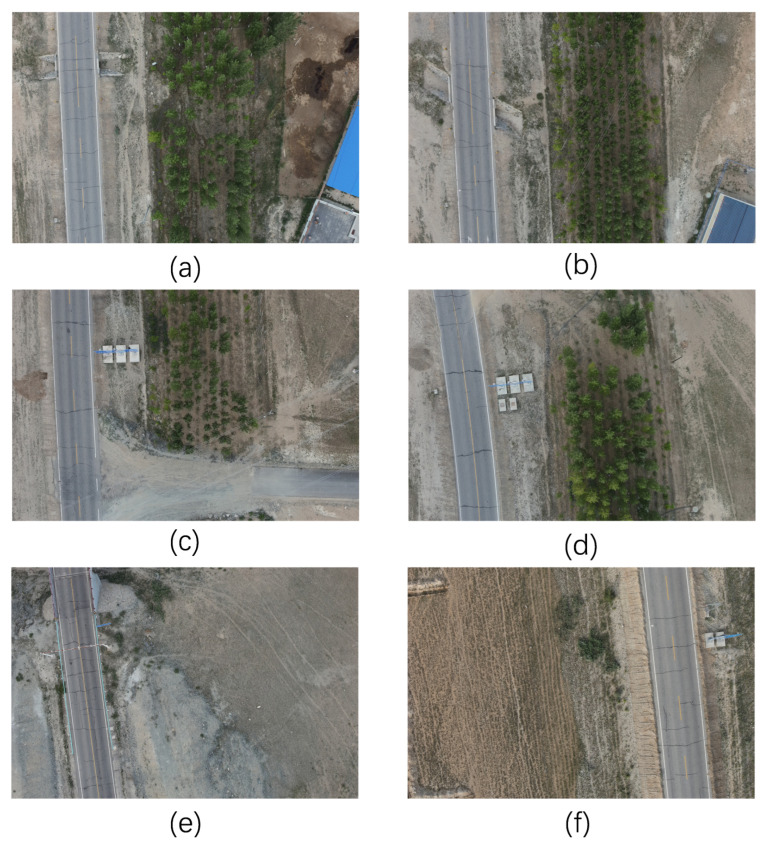
Selected images of infrastructure along the highway. Figures (**a**,**b**) contain culverts and warning stakes; figures (**c**,**d**) show traffic signs; figure (**e**) contains barriers and gantries; figure (**f**) contains traffic signs and shoulder indicators.

The drone is set to fly at a fixed altitude, with a constantly changing view of the target, varying sizes of objects to be detected, and complex noise around the target. Figure 6 shows some of the infrastructure along the road to be detected.

### 4.2. Evaluation Metrics

In practical application scenarios, a single metric cannot comprehensively evaluate the merits of the model. To comprehensively evaluate the performance of the infrastructure detection algorithm along the highway, this paper uses three metrics: precision, recall, and mean Average Precision (mAP).

In the calculation of the target detection evaluation index, the following four parameters are used: TP (True Positive), FP (False Positive), TN (True Negative), FN (False Negative). The specific meanings are shown in Table 2.

In target detection, there are positive and negative samples. Positive samples are image areas where the intersection over union (IOU) with the real box is higher than the IOU threshold (usually set to 0.5); negative samples are image areas where the IOU value with the real box is lower than the IOU threshold.

Precision: Refers to the ratio of the number of positive samples correctly predicted to the number of all samples predicted to be positive, using the following formula:

(8) $$Precision=\frac{TP}{TP+FP}$$

Recall: The ratio of the number of positive samples correctly predicted to the number of true positive samples, which is given by the following formula:

(9) $$Recall=\frac{TP}{TP+FN}$$

Accuracy: The ratio of the amount of data that was correctly predicted to the amount of all data involved in the prediction, calculated as in (10). Accuracy can only be used as a good measure when the values of false positives and false negatives are almost identical.

(10) $$Accuracy=\frac{TP+TN}{TP+FP+TN+FN}$$

AP (Average Precision): The area under the PR smoothing curve for a specific class of all images, with the following formula:

(11) $$AP=\int_{0}^{1}Precision\left(Recall\right)dRecall\;$$

mAP (mean Average Precision): is obtained by taking the combined weighted average of the AP of all categories detected. The formula is as follows:

(12) $$mAP=\frac{1}{N}\sum_{i=1}^{N}AP_{i}$$

where $AP_{i}$ denotes the average precision of the target of class *i*, and *N* denotes the number of targets to be detected in the dataset, and in this paper, N is 8.

### 4.3. Ablation Experiments

In this section, the effectiveness of the various improved modules is experimentally verified to validate the improved performance of the proposed method for the detection of infrastructure datasets along roads based on UAV photography.

Using CenterNet as a baseline, this paper explores the impact of ResNet50, multi-scale feature fusion, and attention mechanisms on the model. The + in Table 3 indicates the hybrid improvement of the module. The baseline network has a precision of 81.5%, a recall of 82.3%, and a mAP of 83.6%. When ResNet50 was used as the backbone network, the mAP improved by 0.6%. When the multi-scale feature fusion module was introduced, more features are retained, and map improves by 1.5% over baseline. The use of an improved attention mechanism solved the problem of too little information after masking and allowed the model to focus more on key features, with a 3.1% increase in mAP over the baseline model.

**Figure 7:**
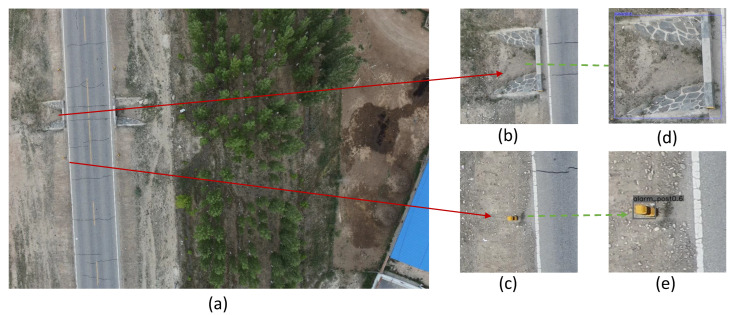
(**a**) The image taken by the UAV; (**b**,**c**) the magnification of the object to be detected; and (**d**,**e**) the effect of the model detection.

The improved model was used to detect infrastructure along the highway. Figure 7 shows the results of the infrastructure detection.

In Figure 7c, the alarm post occupies 52×37 pixels, which is a small percentage compared to the original image of 8192×5460 pixels. The detection precision of alarm posts is improved from 80.1% to 86.5%. The detection performance of this type of very small target has been greatly improved by using an improved multi-scale feature fusion module and adding an attention mechanism.

**Figure 8:**
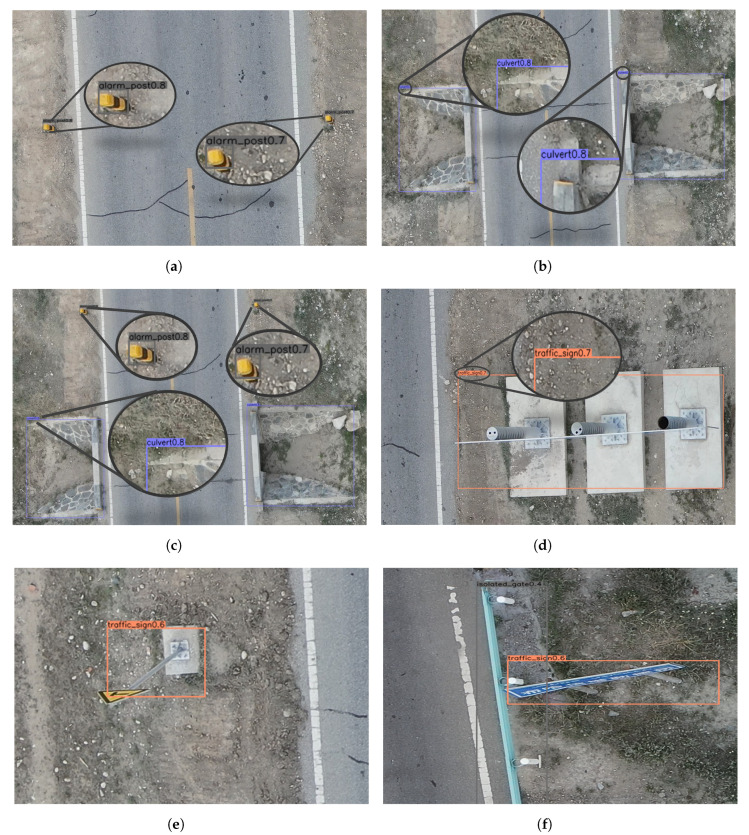
(**a**–**f**) Partial detection results.

Figure 8 shows the results of the detection of infrastructure images along a highway based on UAV aerial photography using the improved algorithm, where the different colored boxes indicate different categories and the value in the top left corner of the box is the confidence level of the corresponding category.

### 4.4. Comparative Experiments

To verify the performance of the algorithms proposed in this paper, in the comparison experiments section, we compared the Faster R-CNN based on RPN (Region Proposal Networks), the SSD algorithm that uses a multi-scale feature map for detection, the RetinaNet model with a focal Loss function, and the one-stage target detection algorithm YOLOv5. The experimental results are shown in Table 4.

Through comparison experiments, we can see that the overall detection effect of our proposed method is better than other target detection algorithms, especially for small targets in the image, such as alarm posts AP, and the milestone MS detection effect is significantly better than other algorithms, we speculate that other detection algorithms are affected by factors such as shooting angle and background environment, resulting in the detector not extracting enough features and thus resulting in the phenomena of missed detection, false detections, etc. Although our method is not as accurate as YOLOv5 for barrier and gantry detection, our improved method achieves 90.7%, 86.5%, 87.7%, 89.6%, 80.2%, and 76.1% detection accuracy for targets such as culverts, alarm posts, traffic signs, milestones, road kerb signs and monitoring facilities, respectively.

## 5. Conclusions

Research results on UAV technology and target detection algorithms continue to develop breakthroughs, and there has been groundbreaking progress in industrial applications. This paper proposes an improved CenterNet target detection model to address the problems of UAV images, such as large variation in target scale, a large number of targets and high proportion of small targets, complex backgrounds, and partially occluded targets to be detected. The model uses a modified ResNet50 as the backbone network to acquire the deep semantic features of image species and introduces a multi-scale feature fusion module to expand the perceptual field through a residual feature fusion layer, which in turn generates a fine-grained feature map. Finally, we introduce an attention mechanism using an inverted residual structure to improve the local feature sensitivity of the model. In addition, we present a dataset of infrastructure along a highway based on UAV photography and validate the improved detection performance of the proposed algorithm on this dataset for targets with large scale variations as well as small targets in images. The mAP value of the proposed algorithm is improved by 3.1% compared to the baseline, and the detection accuracy is also significantly higher than other mainstream target detection algorithms, such as YOLOv5. In the comparative experiments, it is seen that YOLOv5 has good detection precision for larger targets, and in future research work, the algorithm can be improved for this algorithm to improve the detection precision and speed of UAV aerial images.

## Figures and Tables

**Table 1:** Infrastructure datasets along highways.

Dataset	Pictures	Culverts	Alarm Posts	Traffic Signs	Milestones	Isolated Gate	Kerb Sign	Portal Frame	Monitor
Training set	1200	1875	5766	970	418	533	457	472	389
Validation set	150	158	724	135	41	83	62	50	46
Test set	150	293	562	78	72	56	55	65	51
Total	1500	2326	7052	1183	531	672	574	587	486

**Table 2:** Specific meaning.

	Positive	Negative
True	TP (forecast as a positive example; actual as a positive example)	TN (forecast as a negative example; actual as a negative example)
False	FP (forecast as a positive example; actual as a negative example)	FN (forecast as a negative example; actual as a positive example)

**Table 3:** The characteristics of various methods.

Model	Precision/%	Recall/%	mAP/%
Baseline	81.5	82.3	83.6
+ ResNet50	81.8	83.6	84.2
+ ResNet50 + Feature_Fusion	86.7	84.3	85.1
+ Attention	84.2	88.9	84.3
+ ResNet50 + Attention	87.4	88.1	86.3
+ ResNet50 + Feature_Fusion + Attention	89.2	90.6	86.7

**Table 4:** Comparison of experimental results of different algorithms. Expressions for each category in the table: CL for culverts, AP for alarm post, TS for traffic signs, IG for isolated gates, MS for milestones, KS for kerb signs, PF for portal frame, MO for monitoring facilities.The bolded text indicates the best results.

Models	CL	AP	TS	IG	MS	KS	PF	MO	mAP
SSD	81.9	65.1	78.6	63.4	69.5	73.1	78.2	71.4	74.9
Faster R-CNN	83.5	72.8	82.3	64.7	82.2	75.5	76.5	72.7	77.4
RetinaNet	86.7	64.6	81.4	65.6	71.6	73.7	78.8	70.6	78.3
YOLOv5	90.4	79.3	84.8	**73.7**	83.1	74.3	**84.3**	73.8	82.6
**Ours**	**90.7**	**86.5**	**87.7**	68.3	**89.6**	**80.2**	78.5	**76.1**	**86.7**

## Data Availability

Not applicable.
